# Augmented liver inflammation in a microsomal prostaglandin E synthase 1 (mPGES-1)-deficient diet-induced mouse NASH model

**DOI:** 10.1038/s41598-018-34633-y

**Published:** 2018-10-31

**Authors:** Janin Henkel, Charles Dominic Coleman, Anne Schraplau, Korinna Jöhrens, Thomas Siegfried Weiss, Wenke Jonas, Annette Schürmann, Gerhard Paul Püschel

**Affiliations:** 1 University of Potsdam, Institute of Nutritional Science, Department of Nutritional Biochemistry, Nuthetal, Germany; 2 Charité University Hospital Berlin, Institute of Pathology, Berlin, Germany; 3 University Hospital Regensburg, University Children Hospital (KUNO) Regensburg, Regensburg, Germany; 4 German Institute of Human Nutrition, Department of Experimental Diabetology, Nuthetal, Germany; 5 German Center for Diabetes Research (DZD), München-Neuherberg, Germany

## Abstract

In a subset of patients, non-alcoholic fatty liver disease (NAFLD) is complicated by cell death and inflammation resulting in non-alcoholic steatohepatitis (NASH), which may progress to fibrosis and subsequent organ failure. Apart from cytokines, prostaglandins, in particular prostaglandin E_2_ (PGE_2_), play a pivotal role during inflammatory processes. Expression of the key enzymes of PGE_2_ synthesis, cyclooxygenase 2 and microsomal PGE synthase 1 (mPGES-1), was increased in human NASH livers in comparison to controls and correlated with the NASH activity score. Both enzymes were also induced in NASH-diet-fed wild-type mice, resulting in an increase in hepatic PGE_2_ concentration that was completely abrogated in mPGES-1-deficient mice. PGE_2_ is known to inhibit TNF-α synthesis in macrophages. A strong infiltration of monocyte-derived macrophages was observed in NASH-diet-fed mice, which was accompanied with an increase in hepatic TNF-α expression. Due to the impaired PGE_2_ production, TNF-α expression increased much more in livers of mPGES-1-deficient mice or in the peritoneal macrophages of these mice. The increased levels of TNF-α resulted in an enhanced IL-1β production, primarily in hepatocytes, and augmented hepatocyte apoptosis. In conclusion, attenuation of PGE_2_ production by mPGES-1 ablation enhanced the TNF-α-triggered inflammatory response and hepatocyte apoptosis in diet-induced NASH.

## Introduction

Besides its function as a glucostat^1,2^, the liver fulfills central functions in lipid metabolism^3,4^. It recycles lipids from remnant particles, if needed it performs *de novo* lipogenesis from carbohydrates and synthesizes triglyceride-rich VLDL particles for the delivery of fatty acids to peripheral organs, primarily adipose tissue and skeletal muscle. It can oxidize fatty acids to cover its energy needs or produce ketone bodies as an energy source for skeletal muscle and brain. If the fatty acid supply temporarily exceeds the demand, hepatocytes can serve as a physiological transient lipid depository. Under conditions of prolonged nutritional calorie and lipid excess, however, hepatocytes accumulate large amounts of lipids. Non-alcoholic fatty liver disease (NAFLD) with hepatic steatosis ensues. Steatosis may be accompanied by hepatocyte death, inflammation and fibrosis and results in the more severe form of the disease, non-alcoholic steatohepatitis (NASH)^5^. While steatosis is always present in NAFLD, NASH only develops in a subset of patients. In the course of NASH development, resident immune cells of the liver get activated and additional immune cells infiltrate into the tissue. These cells produce and release cytokines as well as small molecule mediators of inflammation, among others prostaglandin E_2_ (PGE_2_). The role of prostaglandins, in particular PGE_2_, in the development of NASH is controversial. Both *in vivo* and *in vitro* evidence suggest that prostaglandins might contribute to the development of steatosis. Thus, both the knockdown of type IV phospholipase A2^6,7^, which releases arachidonic acid for prostaglandin synthesis from phospholipids, or a selective inhibition of cyclooxygenase 2 (COX-2)^8^, the key enzyme in prostaglandin synthesis, protected against diet-induced hepatic steatosis. In addition, prostaglandin E_2_ has been shown to enhance lipid accumulation in hepatocytes by an inhibition of VLDL-synthesis and β-oxidation^9–12^. Kupffer cell-derived PGE_2_ was responsible for lipid accumulation in hepatocytes in alcohol-induced hepatic steatosis^13^. In contrast, PGE_2_ suppressed the expression of enzymes involved in *de novo* fatty acid synthesis in the liver^14^ and hence could protect against steatosis.

Similar controversy exists concerning the role of PGE_2_ in inflammation. Pharmacological or genetic inhibition of PGE_2_ production has been shown to attenuate the inflammatory response in various inflammation models^15–17^ and the inhibition of COX-2 has been shown to inhibit NASH development in type 2 diabetic rats^18^, arguing in favor of a pro-inflammatory impact of PGE_2_. However, PGE_2_ is also known to inhibit the production and release of the pro-inflammatory cytokine tumor necrosis factor α (TNF-α) from macrophages and Kupffer cells via EP2 and EP4 receptors^19,20^. In addition, PGE_2_ significantly inhibited hepatic natural-killer cell activity *in vitro*^21^. Transgenic over-expression of COX-2 in liver partially protected from diet-induced NASH-development and fibrosis^22^. Taken together, the latter data might indicate that PGE_2_ can attenuate hepatic inflammation.

Microsomal PGE synthase 1 (mPGES-1) is assumed to be the key enzyme responsible for the production of the majority of PGE_2_ in the context of inflammation. We could show that it is upregulated in human livers from NASH patients compared to healthy control livers. In order to elucidate the potential role of PGE_2_ in hepatic NASH development, control mice and mice lacking mPGES-1 were fed a cholesterol-containing high-fat diet with a high content of ω6-polyunsaturated fatty acids (PUFA), which has previously been shown to induce NASH in mice^23^. While both control and mPGES-1-deficient mice developed comparable obesity and hepatic steatosis, the inflammatory response including TNF-α production and the ensuing hepatocyte apoptosis was significantly stronger in livers of mPGES-1-deficient mice, indicating that PGE_2_ might attenuate the inflammatory response during NASH development.

## Results

### Up-regulation of key PGE_2_ synthesis enzymes in the liver of NASH patients

**Figure 1:**
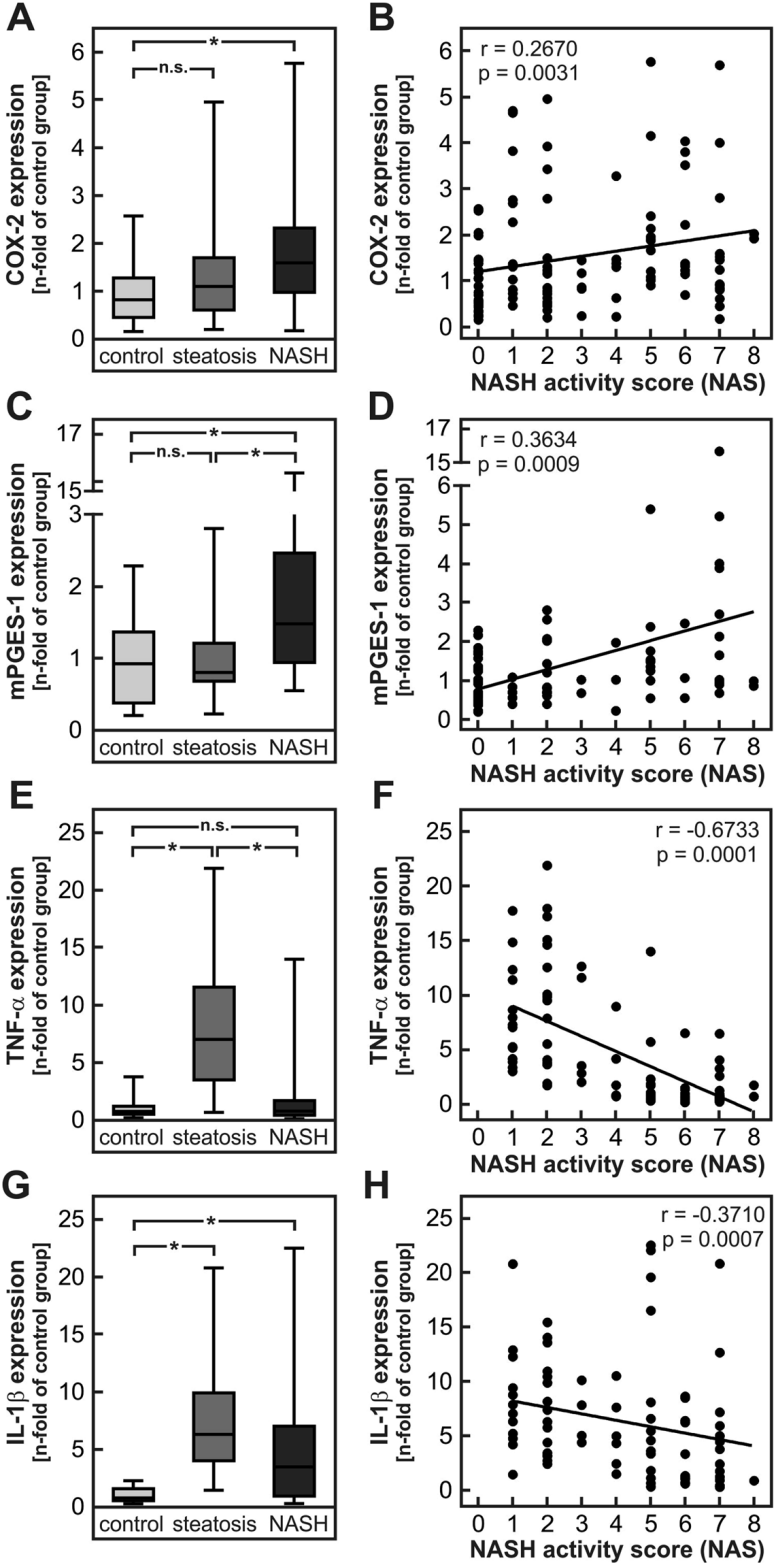
Expression of prostaglandin E_2_ synthesizing enzymes, IL-1β and TNF-α in liver samples of control, steatosis and NASH patients as well as correlation with NASH activity score (NAS). Relative mRNA expression of COX-2 (gene name *PTGS2*) (**A**,**B**), mPGES-1 (gene name *PTGES*) (**C**,**D**), tumor necrosis factor α (TNF-α) (**E**,**F**) and interleukin-1β (IL-1β) (**G**,**H**) were determined in liver samples of a cohort described previously^24,25^. Patient characteristics were summarized in Supplementary Table 1. Values are median (line), upper- and lower quartile (box) and extremes (whiskers) of 27–34 controls, 27–46 patients with hepatosteatosis and 27–43 patients with NASH (**A**–**G**). Single values of COX-2 and mPGES-1 expression were correlated with NAS of controls (NAS < 1), patients with steatosis (1 < NAS ≤ 5) and patients with NASH (NAS > 5) (**B**,**D**) and single values of and IL-1β and TNF-α expression were correlated with NAS of patients with steatosis (1 < NAS ≤ 5) and patients with NASH (NAS > 5) (F, H). Statistics: (**A**–**G**) One-way-ANOVA with Tukey’s post hoc test for multiple comparisons, *p < 0.05; (**B**–**H**) Spearman correlation.

Apart from cytokines, prostaglandins, in particular prostaglandin E_2_ (PGE_2_), play an outstanding role in the control of inflammatory processes. During inflammation, PGE_2_ is formed from the fatty acid arachidonic acid by the subsequent action of the two inducible enzymes prostaglandin-endoperoxide synthase 2 (COX-2, gene name *Ptgs2*) and microsomal prostaglandin E synthase 1 (mPGES-1, gene name Ptges). To test whether PGE_2_ might be a relevant factor for the development of NASH, mRNA expression of these two key synthesis enzymes was determined in a set of histologically confirmed human NASH liver samples in comparison with healthy controls^24,25^ (characteristics of the human study cohort are summarized in Supplementary Table S1). Both COX-2 and mPGES-1 were significantly induced, approximately two-fold higher in NASH livers in comparison with controls or patients with Steatosis (Fig. 1A,C), indicating that the capacity for PGE_2_ synthesis is increased in human NASH livers. Furthermore, mRNA expression of COX-2 and mPGES-1 in the whole study cohort correlated significantly with the NASH activity score (NAS) (Fig. 1B,D). TNF-α expression was induced in patients with steatotic livers but not in livers of NASH patients (Fig. 1E). The expression correlated negatively with the NASH activity score (NAS) in patients with steatosis or NASH (Fig. 1F). IL-1β mRNA was significantly induced in patients with steatosis or NASH compared to healthy controls (Fig. 1G) but IL-1β expression was 20% lower in NASH livers compared to steatosis livers. The expression of the IL-1β mRNA negatively correlated with NASH activity score (NAS) in patients with steatosis or NASH (Fig. 1H). These data indicated that, in accordance with the hypothesis of the existence of a prostaglandin E_2_-dependent negative feedback inhibition loop, the enhanced capacity for PGE_2_ production in NASH patients might reduce TNF-α expression. Subsequently this might result in reduced expression of IL-1β (see discussion below).

### Impairment of PGE_2_-mediated anti-inflammatory feedback loops in mPGES-1-deficient mice

**Figure 2:**
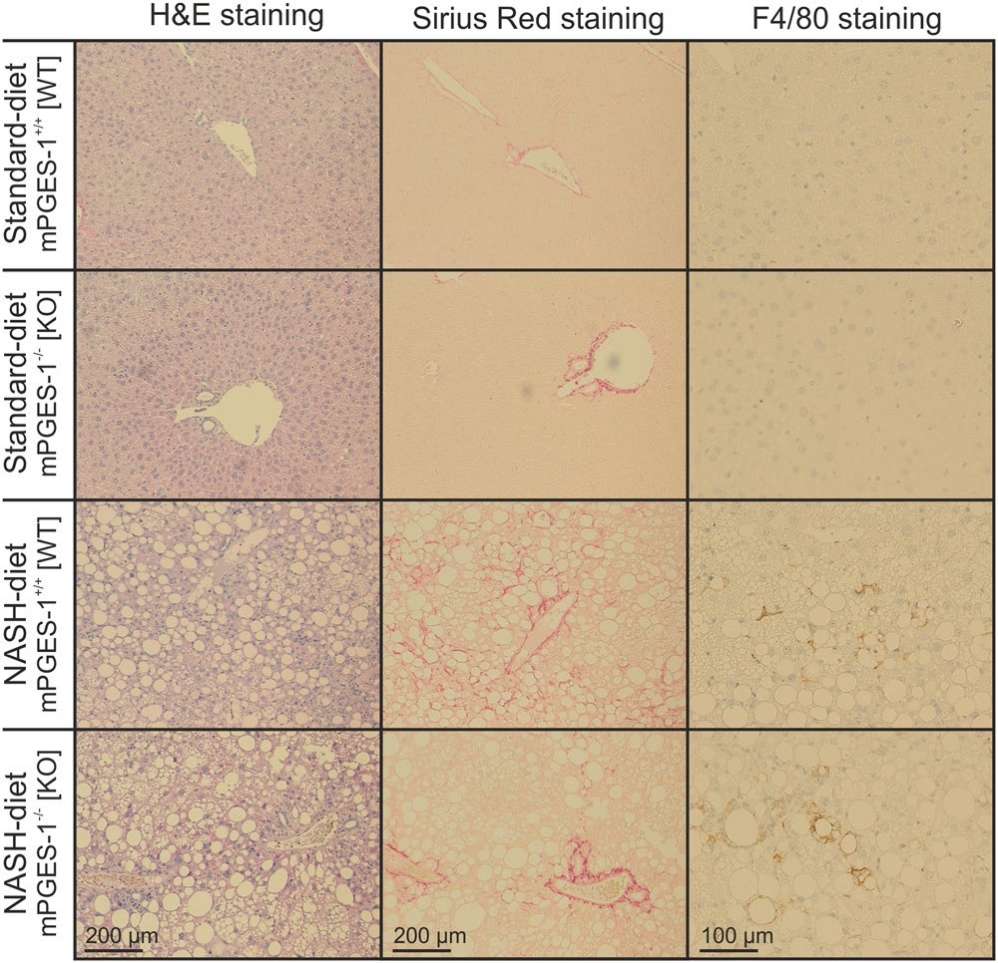
NASH-diet induced steatohepatitis with steatosis, fibrosis and macrophage infiltration in wild-type and mPGES-1-deficient mice. Male mPGES-1^+/+^ (WT) or mPGES-1^−/−^ (KO) mice received the diets for 20 weeks. Representative microphotographs of liver histology, magnification 10× or 20×.

Since it is not possible to undertake mechanistic studies in human liver samples, mice with diet-induced NASH have often been used as alternative models^26^. Male wild-type or mPGES-1-deficient C57BL/6J were fed either chow or a soybean oil-based high-fat diet rich in ω6-polyunsaturated fatty acids and enriched with 0.75% cholesterol (NASH-diet) (Supplementary Table S2) to elucidate the potential impact of PGE_2_ production on inflammation during NASH development. This high-fat diet has recently been shown to induce symptoms of the metabolic syndrome and NASH when fed to mice for 20 weeks (soybean oil + cholesterol-diet,^23^). As previously shown, animals on the NASH-inducing diet developed liver steatosis and early stages of liver fibrosis (Fig. 2). A strong infiltration with macrophages was shown by F4/80 staining and gene expression as a general macrophage marker (Fig. 2, Supplementary Figure S1A and B). Similarly, expression of Cd68 as a marker for resident macrophages and Cd11b as a marker for infiltrating macrophages was induced (Supplementary Figure S1 C and D). No major histological difference in the expression of these macrophage marker genes was observed between wild-type and mPGES-1-deficient mice. The NASH-activity score was elevated to a similar extent in both genotypes after feeding the NASH-diet (Table 1). Most clinical parameters were identical between wild-type and mPGES-1-deficient mice except for insulin resistance score and aspartate aminotransferase (ASAT) activity in serum, which were significantly higher in mPGES-1-deficient mice under NASH diet than in the respective wild-type animals (Supplementary Table S3). The De-Ritis ratio that defined the ration between ASAT and alanine aminotransferase (ALAT) activity as a marker for hepatocellular injury was significantly enhanced in mPGES-1-deficient mice compared to wild type-mice fed a NASH-diet (2.98 ± 0.29 versus 2.08 ± 0.17) indicating that mPGES-1-deficient mice suffered from more severe hepatocyte damage. Hemolysis as potential confounder for enzyme activity in serum analysis could be excluded (Supplementary Figure S2).

**Table 1:** NASH activity score grading steatosis, ballooning (hepatocyte hypertrophy), inflammation and fibrosis in wild-type and mPGES-1-deficient mice.

Scoring parameter	Standard-diet mPGES-1^+/+^ [WT]	Standard-diet mPGES-1^−/−^ [KO]	NASH-diet mPGES-1^+/+^ [WT]	NASH-diet mPGES-1^−/−^ [KO]
Steatosis	0.00 ± 0.00	0.00 ± 0.00	3.88 ± 0.07	3.47 ± 0.26
Hepatocyte hypertrophy	0.00 ± 0.00	0.00 ± 0.00	1.67 ± 0.12	1.47 ± 0.18
Inflammation	0.05 ± 0.05	0.20 ± 0.11	1.58 ± 0.16	1.89 ± 0.20
Fibrosis	0.30 ± 0.11	0.13 ± 0.09	0.96 ± 0.04	1.00 ± 0.00
NASH activity score (NAS)	0.00 ± 0.00	0.00 ± 0.00	8.08 ± 0.21*	7.84 ± 0.44^#^

Values are mean ± SEM of 15–25 mice per group. Statistics: Mann-Whitney-U-Test. *p < 0.05.

*versus STD WT.

^#^versus STD KO.

**Figure 3:**
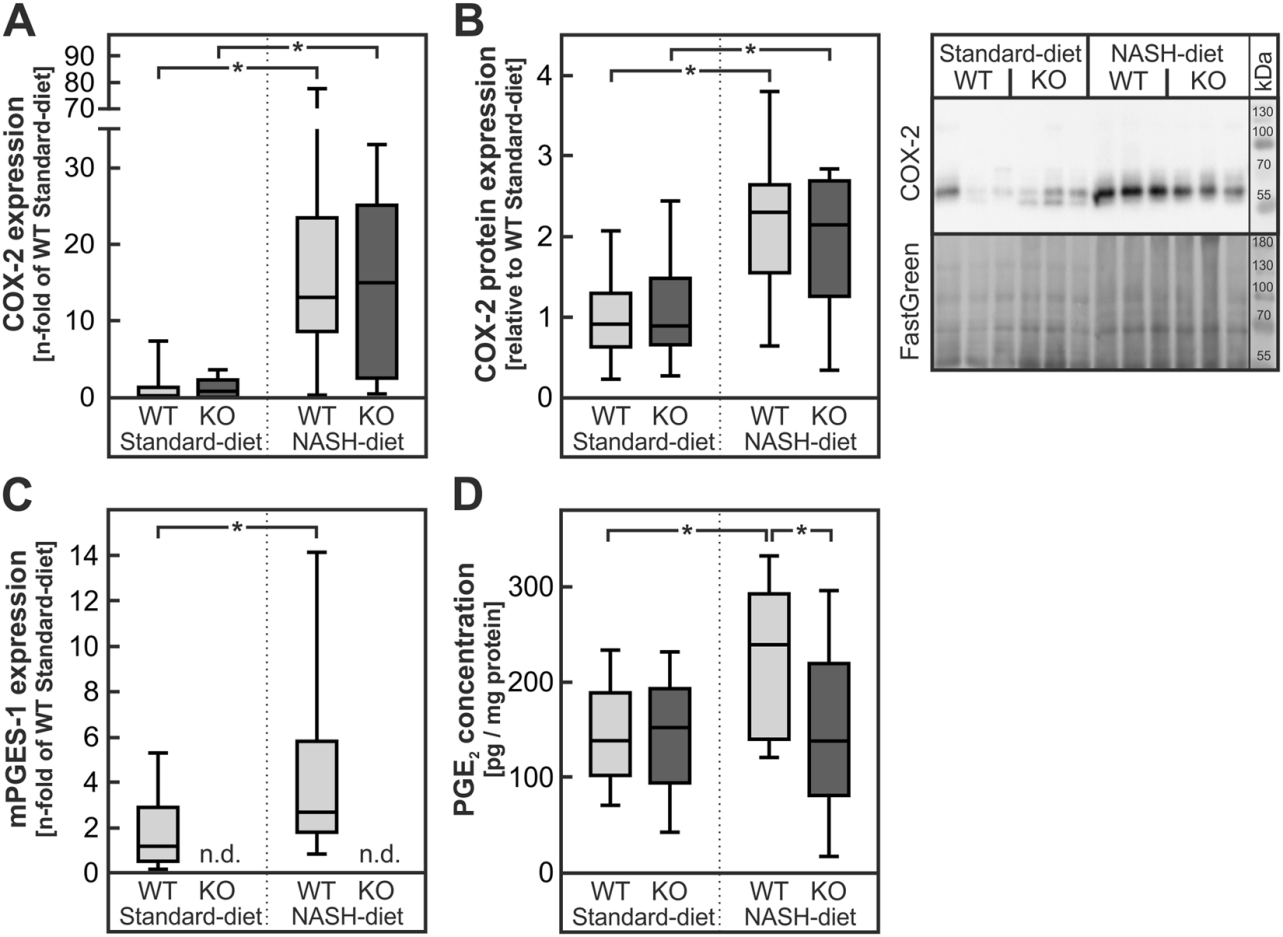
Expression of PGE_2_-synthesising enzymes and PGE_2_ levels in livers of wild-type and mPGES-1-deficient mice fed a standard- or NASH-diet. Male mPGES-1^+/+^ (WT) or mPGES-1^−/−^ (KO) mice received the diets for 20 weeks. (**A**,**B**) Relative mRNA and protein expression of COX-2 (gene name *Ptgs2*) including representative blots in mice liver homogenates. Original blots are provided in Supplementary Figure S3. Dense intensity of COX-2 was normalized to FastGreen staining, which was verified on the same Western blot membrane as a loading control and calculated relative to the group ‘WT Standard-diet’ on each gel. (**C**) Relative mRNA expression of mPGES-1 (gene name *Ptges*). (**D**) PGE_2_ levels were determined in liver homogenates. Values are median (line), upper- and lower quartile (box) and extremes (whiskers) of 12–28 mice per group. Statistics: Two-way-ANOVA with Tukey’s post hoc test for multiple comparisons. *p < 0.05. n.d.: not detectable.

As expected from the results of the human study, COX-2 mRNA was induced about 10-fold by the NASH diet both in wild-type and mPGES-1-deficient animals (Fig. 3A). A significant induction of COX-2 was also determined on the protein level (Fig. 3B, originals blots in Supplementary Figure S3). By contrast, mPGES-1 mRNA was induced 3-fold in wild-type animals but was absent from livers of mPGES-1-deficient mice both under standard- and NASH-diet. As anticipated, the induction of the key enzymes of prostaglandin E_2_ synthesis resulted in significantly increased hepatic PGE_2_ levels in NASH-diet-fed wild-type animals (Fig. 3C). By contrast, PGE_2_ levels in mPGES-1-deficient animals were not affected by the diet (Fig. 3D). Although multiple commercial antibodies were tested in both genotypes, none of these allowed quantification of mPGES-1 expression on the protein level (data not shown).

Depending on the cell type and additional modulating factors, PGE_2_ may either enhance or inhibit the inflammatory response^20,27^. In order to characterize the role of PGE_2_ in the development of diet-induced NASH, potential downstream targets of PGE_2_ were analyzed in wild-type and mPGES-1-deficient mice.

**Figure 4:**
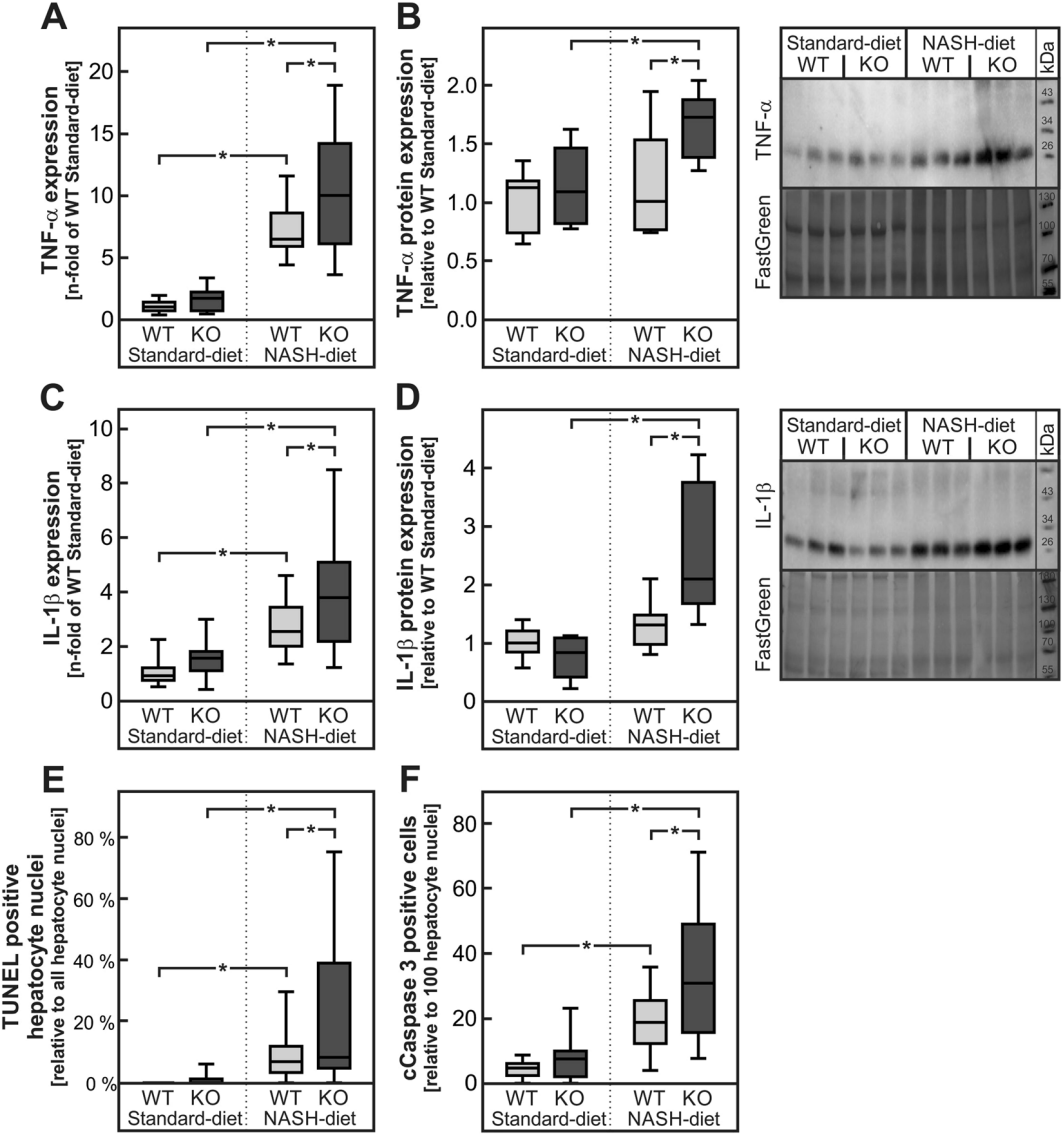
Expression of TNF-α and IL-1β and quantification of apoptosis in livers of wild-type and mPGES-1-deficient mice fed a standard- or NASH-diet. Male mPGES-1^+/+^ (WT) or mPGES-1^−/−^ (KO) mice received the diets for 20 weeks. (**A**,**B**) Relative mRNA and protein expression of TNF-α. (**C**,**D**) Relative mRNA and protein expression of IL-1β. Dense intensity of TNF-α (**B**) or IL-1β (**D**) was normalized to FastGreen staining, which was verified on the same Western blot membrane as a loading control and calculated relative to the group ‘WT Standard-diet’ on each gel. Original blots are provided in Supplementary Figure S4 and S5. (**E**,**F**) Quantification of hepatocyte apoptosis by TUNEL assay and immunohistochemistry staining of cleaved caspase 3 (cCaspase 3) by calculating the number of TUNEL- or cleaved caspase 3-positive cells relative to the number of hepatocyte nuclei per field in 5 randomly chosen microphotographs per liver section with 5–6 livers per group. Values are median (line), upper- and lower quartile (box) and extremes (whiskers) of 15–28 (**A**,**C**) or 7–9 (**B**,**D**) mice or 25–30 samples (**E**,**F**) per group. Statistics: Two-way-ANOVA with Tukey’s post hoc test for multiple comparisons. *p < 0.05.

The mRNA level of the pro-inflammatory cytokine tumor necrosis factor α (TNF-α) was increased in livers of NASH-diet-fed wild-type and mPGES-1-deficient mice in comparison to the respective standard-diet-fed controls (Fig. 4A). PGE_2_ is known to inhibit the induction of TNF-α in macrophages^20^. Therefore we hypothesized that the lack of PGE_2_ in mPGES-1-deficient mice might result in an enhanced diet-induced TNF-α expression in these livers. In line with this hypothesis, the diet-dependent induction of hepatic TNF-α mRNA was significantly more pronounced in mPGES-1-deficient mice than in wild-type animals (Fig. 4A). A similar pattern of induction was observed on the protein level: TNF-α protein expression increased only slightly but not significantly in wild-type NASH-diet-fed animals but was significantly increased in NASH-diet-fed mPGES-1-deficient mice (Fig. 4B, original blots in Supplementary Figure S4).

Alike TNF-α, the mRNA of the pro-inflammatory cytokine interleukin-1β (IL-1β) was induced in the livers of NASH-diet-fed animals (Fig. 4C). In contrast to TNF-α, the expression of IL-1β can be increased by PGE_2_^28^. The diet-dependent IL-1β induction was therefore expected to be lower in mPGES-1-deficient mice. However, IL-1β mRNA levels were significantly higher in NASH-diet-fed mPGES-1-deficient mice than in the corresponding wild-type group (Fig. 4C). Similar results were obtained on the protein level: the IL-1β protein level increased only slightly but not significantly in wild-type mice fed a NASH-diet but by contrast, a significant two-fold increase in IL-1β protein was observed in NASH-diet-fed mPGES-1-deficient mice (Fig. 4D, original blots in Supplementary Figure S5).

TNF-α is known to enhance hepatocyte apoptosis, which was analyzed by Terminal deoxynucleotidyl transferase dUTP Nick End Labeling (TUNEL) assay as well as immunohistochemically staining of cleaved caspase 3. In livers of mice fed the NASH-diet the number of apoptotic hepatocytes was increased (Fig. 4E,F). Furthermore, the number of TUNEL- or cleaved caspase 3-positive cells was significantly higher in livers of mPGES-1-deficient mice compared to wild type mice fed the NASH-diet (Fig. 4E,F).

### Cellular components involved in the differences of the PGE_2_-dependent changes in the inflammatory response

**Figure 5:**
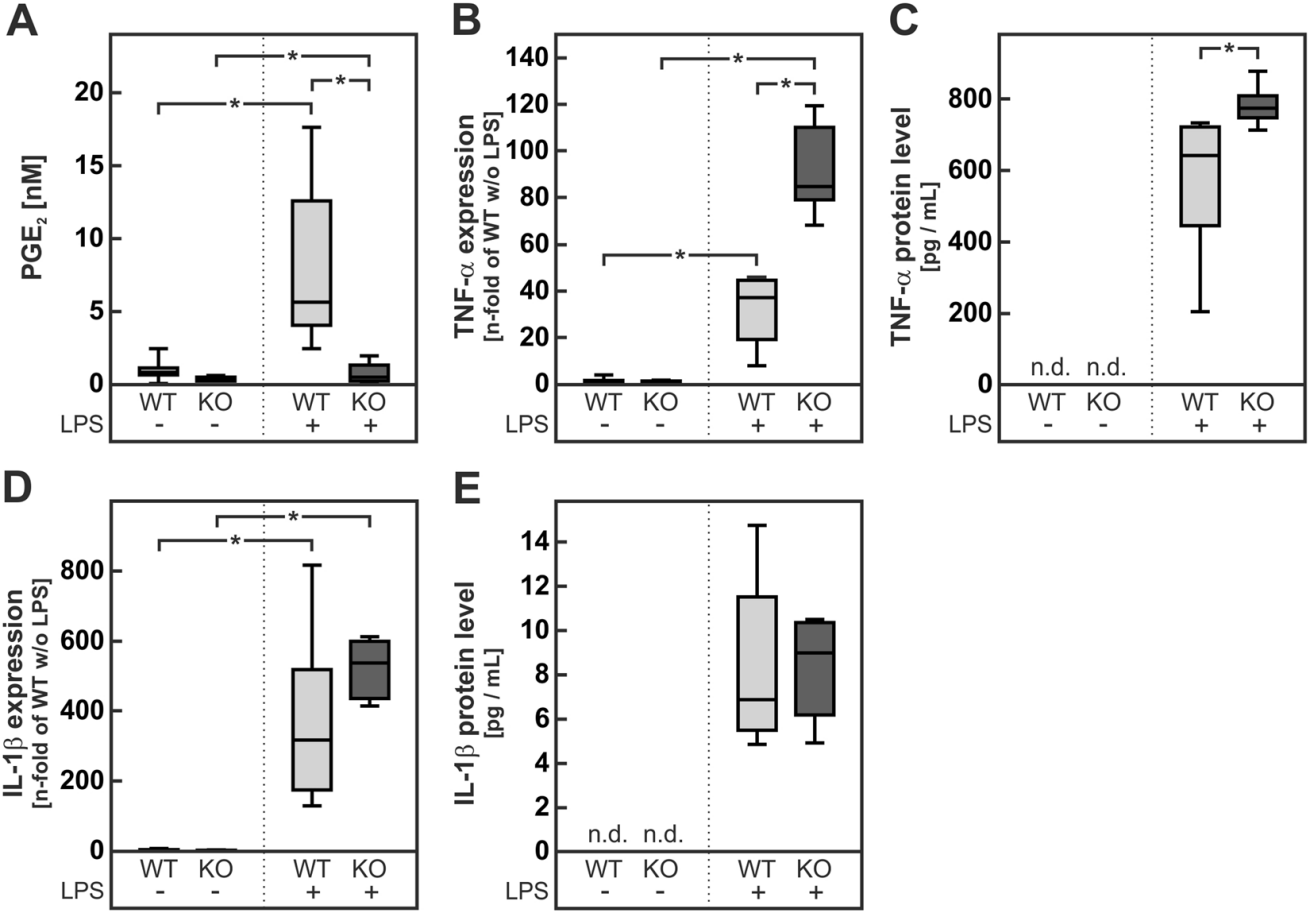
PGE_2_ levels, TNF-α and IL-1β expression in isolated peritoneal macrophages from wild-type and mPGES-1-deficient mice. Primary peritoneal macrophages from male mPGES-1^+/+^ (WT) or mPGES-1^−/−^ (KO) mice were stimulated with 1 ng/mL LPS for 24 h. Levels of PGE_2_ (**A**), TNF-α (**C**) and IL-1β (**E**) were determined in cell culture supernatants. Relative mRNA expression of TNF-α (**B**) and IL-1β (**D**). Values are median (line), upper- and lower quartile (box) and extremes (whiskers) of 6 independent experiments. Statistics: Two-way-ANOVA with Tukey’s post hoc test for multiple comparisons. *p < 0.05. Abbreviations: LPS: lipopolysaccharide; n.d.: not detectable.

Resident macrophages (Kupffer cells) and newly recruited infiltrating macrophages are among the potential hepatic sources of pro-inflammatory cytokines and PGE_2_. To determine, whether an alteration of PGE_2_ and cytokine production in macrophages of mPGES-1-deficient mice is responsible for the enhanced NASH diet-induced TNF-α and IL-1β production in these animals, peritoneal macrophages were isolated from these animals and stimulated with LPS in culture. Peritoneal macrophages isolated from chow-fed wild-type and mPGES-1-deficient mice did not secrete detectable amounts of PGE_2_ into the cell culture supernatant (Fig. 5A). After stimulation with LPS, PGE_2_ production was induced only in wild-type peritoneal macrophages whereas there was no enhanced production in mPGES-1-deficient peritoneal macrophages (Fig. 5A). LPS induced TNF-α expression and increased TNF-α secretion into the medium. Both induction and secretion were significantly higher in mPGES-1-deficient macrophages than in wild-type cells (Fig. 5B,C). Apparently the postulated autocrine PGE_2_-mediated feedback inhibition loop was operative in peritoneal macrophages.

**Figure 6:**
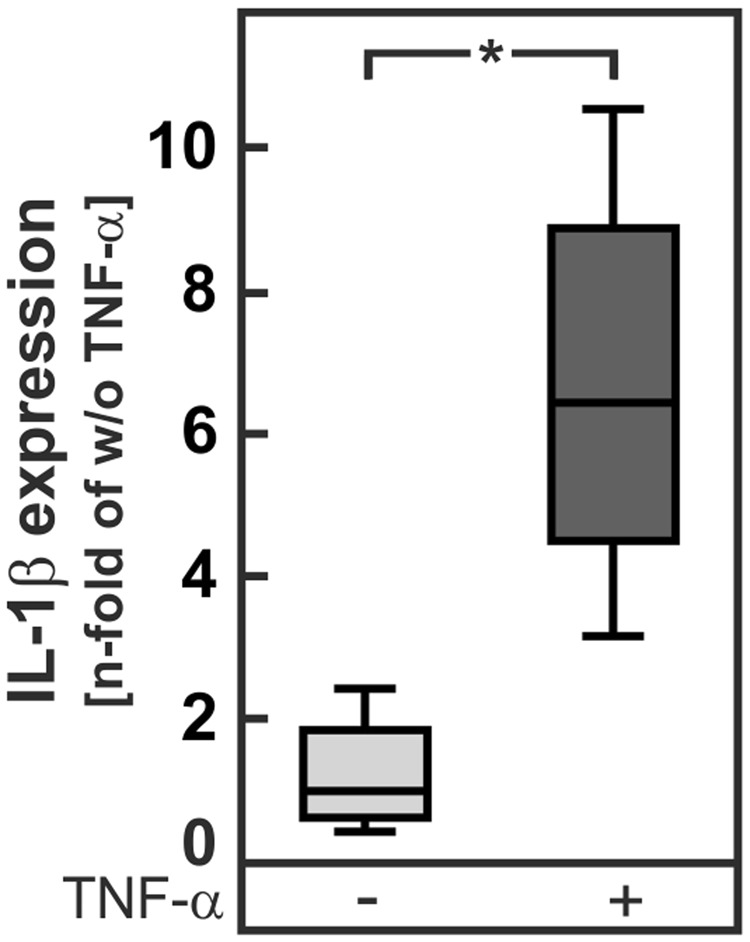
IL-1β expression in isolated hepatocytes from wild-type mice. Primary hepatocytes from male mPGES-1^+/+^ (WT) mice were stimulated with 30 ng/mL TNF-α for 6 h. Relative mRNA expression of IL-1β. Values are median (line), upper- and lower quartile (box) and extremes (whiskers) of 5 independent experiments. Statistics: Student’s t-test for unpaired samples. *p < 0.05.

In contrast to the TNF-α production, IL-1β expression and secretion in peritoneal macrophages was not affected by PGE_2_ (Fig. 5D,E). Therefore, it was assumed that the enhanced IL-1β formation in livers of NASH-diet-fed mPGES-1-deficient mice (Fig. 4C,D) might be due to a TNF-α-triggered IL-1β formation in hepatocytes. In accordance with this assumption, TNF-α strongly induced IL-1β expression in hepatocytes (Fig. 6).

## Discussion

The current study showed that the hepatic expression of enzymes for PGE_2_ synthesis increased with the severity of NASH in patients (Fig. 1) as well as in a diet-induced mouse NASH model (Fig. 3). Deficiency of mPGES-1, a key enzyme for inducible PGE_2_ synthesis, in this mouse model augmented the inflammatory response. It abolished the diet-induced increase in hepatic PGE_2_ levels, thereby disrupting a feedback inhibition loop that limits the production of TNF-α in infiltrating macrophages. The resulting increase in TNF-α enhanced the IL-1β formation, most likely in hepatocytes, as well as hepatocyte apoptosis (Figs 4, 6).

**Figure 7:**
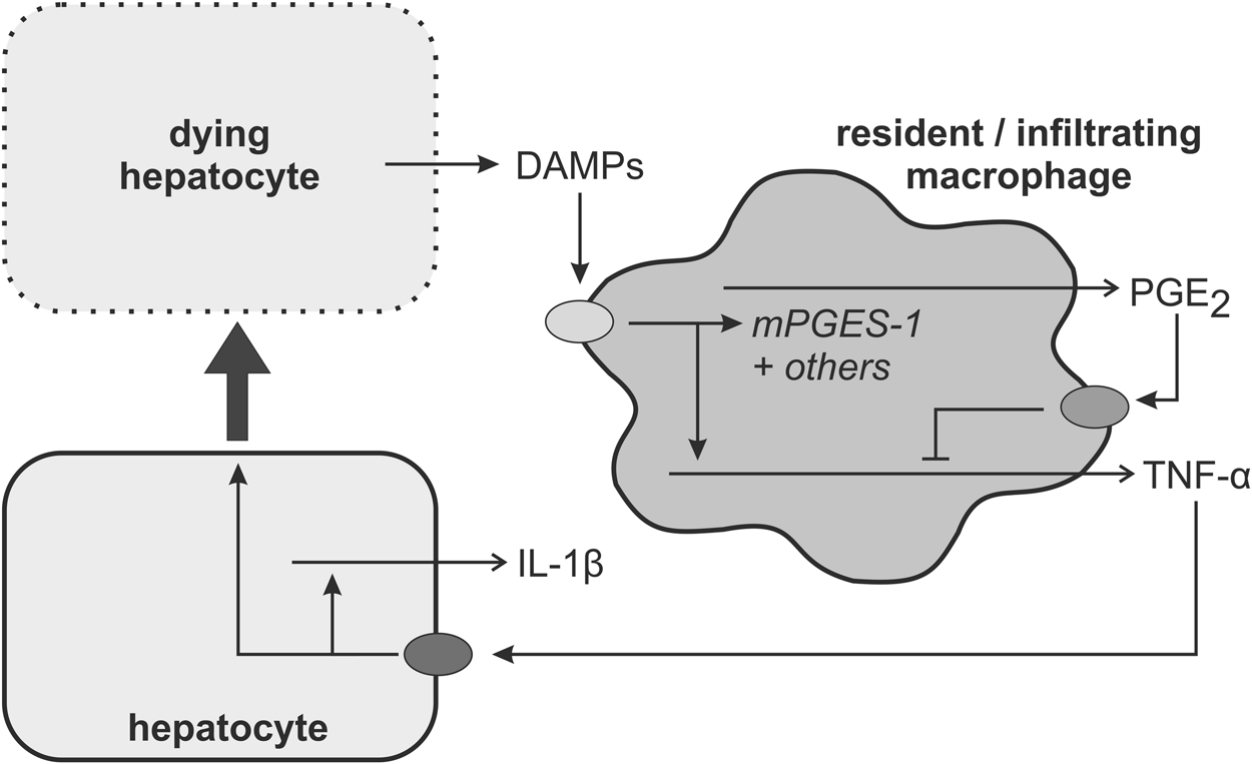
Schematic overview. See main text for details. Abbreviations: DAMPs: damage associated molecular pattern; IL-1β: interleukin-1β; PGE_2_: prostaglandin E_2_; mPGES-1: microsomal PGE synthase 1; TNF-α: tumor necrosis factor α.

In the course of NASH, dying hepatocytes release damage-associated molecular patterns (DAMPs) that may trigger an inflammatory response in macrophages. While in the early phase of NASH development Kupffer cells appear to be the most relevant macrophage population, in later phase infiltrating monocyte-derived macrophages are pivotal for the progression of NASH^29^. Upon stimulation with DAMPs, macrophages release pro-inflammatory cytokines, among others TNF-α, as well as PGE_2_ via an induction of mPGES-1 (Fig. 7). PGE_2_ inhibited TNF-α production in peritoneal macrophages with an IC_50_ of 7 nM (Supplementary Figure S6). Thus, PGE_2_ produced in macrophages in response to pro-inflammatory stimuli can inhibit the stimulus-dependent TNF-α formation in an autocrine negative feedback inhibition loop. This feedback inhibition loop was disrupted in macrophages of mPGES-1-deficient mice resulting in an enhanced TNF-α production. TNF-α can act on hepatocytes where it induced the production of IL-1β. This explains why IL-1β levels were increased in NASH livers of mPGES-1-deficient mice although PGE_2_ has been shown to enhance IL-1β production in macrophages^30^. These results are in accordance with the correlative data obtained in the human study (Fig. 1). In addition, the increase in hepatic TNF-α levels also promote hepatocyte death thereby further increasing the DAMP-dependent progression of the inflammation (Figs 4 and 7). Therefore, elimination of the PGE_2_-dependent feedback inhibition loop in macrophages augmented the TNF-α-dependent inflammatory response in the liver. mPGES-1-deficiency in hepatocytes on diet-induced hepatic TNF-α induction could be excluded because no genotype-specific differences in hepatic TNF-α mRNA levels were observed in livers of NASH-diet-fed mice with a hepatocyte-specific deficiency of cyclooxygenase 2 (Cox-2), the second key enzyme of induced PGE_2_ generation that acts upstream of mPGES-1 (preliminary unpublished data). A potential impact of mPGES-1 deficiency in extrahepatic organs on the hepatic TNF-α formation although unlikely cannot be excluded in the current model.

Although TNF-α-mediated hepatocyte apoptosis was significantly increased in mPGES-1-deficient mice compared to wild- type mice fed a NASH-diet (Fig. 4E,F), no genotype-specific differences in macrophage infiltration or fibrosis could be observed (Fig. 2 and Supplementary Figure S1). This may be due either to the fact that the feeding intervention of 20 weeks was not long enough to allow the development of more advanced stages of the disease or by the fact that compensatory repair mechanisms were simultaneously triggered and might prevent a profound change in histology.

These findings have a potential clinical implication: Key PGE_2_-synthetic enzymes were induced in human NASH livers (Fig. 1) enhancing the local PGE_2_ production in the liver. PGE_2_ clearly attenuated the TNF-α-mediated inflammatory response, indicating that pharmacological inhibition of its production might potentially be harmful in NASH patients.

A similar mPGES-1-mediated PGE_2_-dependent attenuation of an inflammatory response was observed in spinal cord inflammation induced by LPS. LPS priming to induce mPGES-1 and PGE_2_ production attenuated subsequent LPS-induced TNF-α and IL-1β formation in wild-type but not in mPGES-1-deficient mice^31^. In a different model, injection of LPS into the peritoneum of mPGES-1-deficient mice resulted in an afebrile inflammatory response that was accompanied by an increase in IL-1β, TNF-α and IL-6. In accordance with the current findings, the LPS-induced cytokine induction was more pronounced in several areas of the brain of knockout mice compared to the wild-type animals, however, at variance with the current findings, LPS-induced TNF-α expression in liver was strongly reduced in knockout mice^32^. In further support of an anti-inflammatory role of PGE_2_ methionine choline-deficient diet-induced TNF-α production was attenuated in livers of animals with a liver-specific over-expression of COX-2, the other inducible key enzyme in PGE_2_ synthesis^22^.

Although previous *in vitro* experiments suggested that PGE_2_ might directly impact on lipid accumulation in hepatocytes^9–12^, the current study did not provide any evidence that elimination of mPGES-1 affected the diet-induced steatosis in NASH-diet-fed mouse livers. No genotype effect was observed on triglyceride or cholesterol accumulation (Supplementary Table S3).

NASH-diet-fed animals developed insulin resistance. This insulin resistance was significantly more pronounced in mPGES-1-deficient mice than in the corresponding wild-type group (Supplementary Table S3). TNF-α is known to interrupt the insulin receptor signal transduction by an inhibitor of κB kinase (IKK)-dependent inhibitory serine phosphorylation of insulin receptor substrates that results in subsequent proteasomal degradation. Thus, the observed increase in TNF-α in mPGES-1-deficient mice might contribute to the enhanced insulin resistance. A direct proof of an impact of the elevated TNF-α levels on hepatic insulin signaling is lacking. In the current investigation, animals with a global mPGES-1 knockout were studied. It can therefore not be excluded that impaired PGE_2_ production in other insulin-sensitive organs, e.g. skeletal muscle or adipose tissue, might be the cause of the greater insulin resistance in mPGES-1-deficient mice.

## Methods

All chemicals were of analytical or higher grade and obtained from local providers unless otherwise stated.

### Human Studies

Gene expression was determined by qPCR in cDNAs from liver tissue samples from control and NAFLD patients described in previous studies^24,25^. The studies were approved by the Ethics commission of the local ethical committees of the University Hospital Regensburg and University Hospital Tübingen, Germany (215/2006V). Informed consent was obtained from all participants.

### Animals

Mice with global deletion of mPGES-1^33^ backcrossed on C57BL/6J were provided by Pfizer. Feeding studies were performed with F2 generation homozygous mPGES-1^+/+^ (WT) and mPGES-1^−/−^ (KO) mice that were generated by breeding homozygous F1 offspring of common heterozygous parents. Genotypes were identified by PCR analysis (Supplementary Figure S7).

### Experimental design and *in vivo* experiments

Wild-type and mPGES-1-deficient mice were randomly assigned to standard or NASH-diet (Supplementary Table S2) for 20 weeks as described previously^23^. Oral glucose tolerance test was performed in week 18 after an overnight fast. Mice were killed by cervical dislocation after isoflurane anesthesia. Serum and organs were snap-frozen in liquid nitrogen and stored at −70 °C for biochemical analysis, aliquots of the organs were fixed for histological examination. Animal experiments were performed according to the ARRIVE guidelines. Treatment of the animals followed the German animal protection laws and was performed with approval of the state animal welfare committee (LUGV Brandenburg, V3-2347).

### Serum and tissue analysis

Serum parameters were quantified by an automated analyzer (Cobas Mira S, Hoffmann-La Roche, Basel, Switzerland) with the appropriate commercially available reagent kits. Liver triglycerides and cholesterol were determined by TRIGS-assay (Randox; Crumlin, UK) and cholesterol liquicolor (HUMAN, Wiesbaden, Germany), respectively.

### Hepatic histology

Formalin-fixed and paraffin-embedded liver sections (2-3 µm) were stained with Hematoxylin & Eosin or Sirius Red (both Sigma-Aldrich, Taufkirchen, Germany). Immunohistochemistry analyses were performed with anti-F4/80 antibody (AbD Serotec, Bio-Rad, Munich, Germany) and cleaved caspase 3 antibody (Cell Signaling Technology, Frankfurt am Main, Germany). Terminal deoxynucleotidyl transferase dUTP Nick End Labeling (TUNEL) assay was achieved with the Click-iT™ TUNEL Colorimetric IHC Detection Kit (Thermo Fisher Scientific, Berlin, Germany). Histological steatosis, inflammation and fibrosis were graded according to the NASH activity score (NAS)^34,35^ by a liver pathologist (KJ) blinded to the diet. Histological staining of F4/80, cleaved caspase 3 and TUNEL-positive cells was by using ImageJ software (version ImageJ 1.51j8, Wayne Rasband, National Institutes of Health, USA) in images of 5 randomly chosen fields of each liver. Details were described in the Supplementary Methods Section in the Supplements.

### Real-time RT-PCR analysis

RNA isolation, reverse transcription and qPCR were performed as previously described^36^. Results are expressed as relative gene expression normalized to expression levels of reference genes (Hprt, Eef2 and Srsf4 in the mice study, YWHAZ in the human study and Hprt in the cell culture experiments) according to the formula: fold induction = 2^(a-b) gene of interest^/2^(a-b) reference gene(s)^. Parameter “a” means the arithmetic mean of all Ct-values from samples of the control group (Standard-diet WT in the mice study and untreated samples in the other experimental data) and parameter “b” means the Ct-value of every single sample. For calculations with more than one reference gene the geometric mean of the difference (a-b) of each reference gene was used.

### Western blot analysis

Western blot was performed as described previously^37^ with anti-COX-2, anti-TNF-α and anti-IL-1β antibodies (Santa Cruz Biotechnology, Heidelberg, Germany) as well as FastGreen-staining (Sigma-Aldrich, Taufkirchen, Germany) as a loading control. Visualization of immune complexes was performed by using chemoluminescence reagent in ChemiDoc™ Imaging System with ImageLab software (Bio-Rad, Munich, Germany).

### Determination of PGE_2_, TNF-α and IL-1β

Cell culture supernatants or liver homogenates in 0.1 M phosphate buffer with 1 mM EDTA and 10 µM indomethacin (both Sigma-Aldrich, Taufkirchen, Germany) were analyzed with enzyme-linked immunoassay kits for determination of PGE_2_ (Cayman Chemical, Ann Arbor, Michigan, USA), TNF-α or IL-1β (both Life Technologies, Darmstadt, Germany) according to the manufacturer’s instructions.

### Isolation, cultivation and treatment of murine hepatocytes and peritoneal macrophages

Cells were isolated from chow-fed male wild-type or mPGES-1-deficient C57BL/6J mice as previously described^20,23,37^. Percoll-purified hepatocytes were cultured for 24 h in Williams E medium (Sigma-Aldrich, Taufkirchen, Germany) containing 1% antibiotics, 100 nM dexamethasone and 0.5 nM insulin (Sigma-Aldrich, Taufkirchen, Germany) as well as 4% fetal calf serum for the first 2 h. Subsequently hepatocytes were stimulated with 30 ng/mL TNF-α (PeproTech, Hamburg, Germany) for 6 h. Peritoneal macrophages were isolated by peritoneal lavage^38^ with 3% fetal calf serum in phosphate-buffered saline and cultured for 24 h in low-endotoxin RPMI medium containing 1% antibiotics and 10% fetal calf serum, as well as 100 ng/mL phorbol-12-myristate-13-acetate (Sigma-Aldrich, Taufkirchen, Germany) for the first 2 h. Macrophages were stimulated with 1 ng/mL lipopolysaccharide (LPS) from *Escherichia coli* (Sigma-Aldrich, Taufkirchen, Germany) for 24 h.

### Statistical analysis

The statistical significance of differences was determined by Student’s t-test for unpaired samples, two-way-ANOVA with Tukey’s post hoc test for multiple comparisons or Mann-Whitney-U-Test for non-parametric samples as detailed in the legends to the figures using GraphPad Prism version 6 for Windows (GraphPad Software, La Jolla California USA). Differences with a p ≤ 0.05 were considered statistically significant.

## Electronic supplementary material

Supplementary Material

## Data Availability

The datasets generated and analyzed during the current study are available from the corresponding author on reasonable request.
