# Supplementary material for: Augmented liver inflammation in a microsomal prostaglandin E synthase 1 (mPGES-1)-deficient diet-induced mouse NASH model
Source: Sci Rep. 2018 Oct 31;8:16127. doi: 10.1038/s41598-018-34633-y (PMC6208405; doi:10.1038/s41598-018-34633-y)
Supplement: Supplementary file 1 — Supplementary Material [file 41598_2018_34633_MOESM1_ESM.pdf]

Augmented liver inflammation in a microsomal prostaglandin E synthase 1 (mPGES-1)-deficient diet-induced mouse NASH model

Janin Henkel, Charles Dominic Coleman, Anne Schraplau, Korinna Jöhrens, Thomas Siegfried Weiss, Wenke Jonas, Annette Schürmann and Gerhard Paul Püschel

Supplementary Figures & Tables

Supplementary Table S1: Summarized characteristics of the human study cohort. Study design and patients were described previously in detail [24,25]. Values are mean ± SEM or numbers of patients. Statistics: One-way-ANOVA with Tukey's or Dunn's (non-parametric data) post hoc test for multiple comparisons, #: $p < 0.05$ versus control group, §: $p < 0.05$ versus steatosis group.

Abbreviations: ALAT: alanine aminotransferase; ASAT: aspartate aminotransferase; CHE: choline esterase.

	Control group	Steatosis group	NASH group
**Sex: Males/ Females**	16 / 18	21 / 25	25 / 18
**Age [years]**	53.9 ± 2.6	60.0 ± 1.7	60.2 ± 2.2
**Body mass index (BMI) [kg/m^2]**	24.8 ± 0.6	29.5 ± 0.8 #	29.6 ± 1.0 #
**Obesity: no obesity**	18 out of 34	12 out of 46	10 out of 43
**pre-obesity**	13 out of 34	18 out of 46	16 out of 43
**obesity grade I**	3 out of 34	11 out of 46	12 out of 43
**obesity grade II**		2 out of 46	3 out of 43
**obesity grade III**		3 out of 46	2 out of 43
**Type II Diabetes**	0 out of 34	6 out of 43	10 out of 43
**NASH activity score (NAS)**	0 ± 0	2 ± 0.2 #	6 ± 0.1 # §
**Enzyme activity in serum: ALAT [U/L]**	22.3 ± 1.7	39.9 ± 5.4	47.5 ± 10.4 #
**ASAT [U/L]**	21.1 ± 1.6	30.1 ± 3.8	54.1 ± 10.5 (#, p = 0.058)
**CHE [U/L]**	7624 ± 373	9473 ± 571 #	9838 ± 421 #

Supplementary Table S2: Diet composition.

	Standard-diet	NASH-diet
**Metabolizing energy [kcal/kg]**	3057	4637
**Carbohydrates [g/kg]**	500	398
**Protein [g/kg]**	190	188
**Fat [g/kg]**	33	255
**Cholesterol [%]**	0.00	0.75

Supplementary Table S3: NASH-diet-dependent changes in body weight and clinical parameters in serum and liver of wild-type and mPGES-1-deficient mice. Male mPGES-1$^{+/+}$ (WT) or mPGES-1$^{-/-}$ (KO) mice received the diets for 20 weeks. Body weight was determined weekly. Relative insulin resistance was calculated by the sum of the products of insulin concentration × glucose concentration during oral glucose tolerance test. Immediately after sacrifice cardiac blood was taken for the analysis of serum parameters. Triglycerides and cholesterol content were analyzed in liver tissue homogenates. Values are mean ± SEM of 18-28 mice per group (except insulin resistance index, 6-7 mice per group). Statistics: Two-way-ANOVA with Tukey's post hoc test for multiple comparisons. *: $p < 0.05$. Abbreviations: ALAT: alanine aminotransferase; ASAT: aspartate aminotransferase.

	**Standard-diet mPGES-1$^{+/+}$ [WT]**	**Standard-diet mPGES-1$^{-/-}$ [KO]**	**NASH-diet mPGES-1$^{+/+}$ [WT]**	**NASH-diet mPGES-1$^{-/-}$ [KO]**
Physiological data				
**Body weight gain [g]**	10.53 ± 0.47	10.26 ± 0.82	15.89 ± 0.74<br>* versus STD WT	16.87 ± 1.08<br>* versus STD KO
**Fat mass [% of body weight]**	13.45 ± 1.03	9.60 ± 1.06	26.69 ± 1.19<br>* versus STD WT	27.41 ± 1.47<br>* versus STD KO
**Insulin resistance index [relative to WT Standard-diet]**	1.00 ± 0.12	0.94 ± 0.13	1.71 ± 0.14<br>* versus STD WT	2.31 ± 0.88<br>* versus STD KO<br>* versus NASH-diet WT
Triglycerides				
**Serum triglycerides [mmol/L]**	1.02 ± 0.05	1.14 ± 0.08	0.56 ± 0.04<br>* versus STD WT	0.62 ± 0.03<br>* versus STD KO
**Liver triglycerides [mg/mg protein]**	0.42 ± 0.05	0.26 ± 0.03	2.21 ± 0.20<br>* versus STD WT	2.42 ± 0.23<br>* versus STD KO
Cholesterol				
**Serum cholesterol [mmol/L]**	1.97 ± 0.07	2.23 ± 0.08	3.97 ± 0.14<br>* versus STD WT	4.05 ± 0.25<br>* versus STD KO
**Liver cholesterol [mg/mg protein]**	0.80 ± 0.04	0.66 ± 0.05	6.14 ± 0.25<br>* versus STD WT	5.63 ± 0.33<br>* versus STD KO
Liver enzyme activity in serum				
**ALAT [U/L]**	24.23 ± 1.41	26.84 ± 1.35	44.60 ± 4.47<br>* versus STD WT	45.03 ± 4.86<br>* versus STD KO
**ASAT [U/L]**	76.35 ± 8.79	76.00 ± 9.08	86.40 ± 6.32	117.95 ± 10.23<br>* versus STD KO<br>* versus NASH-diet WT

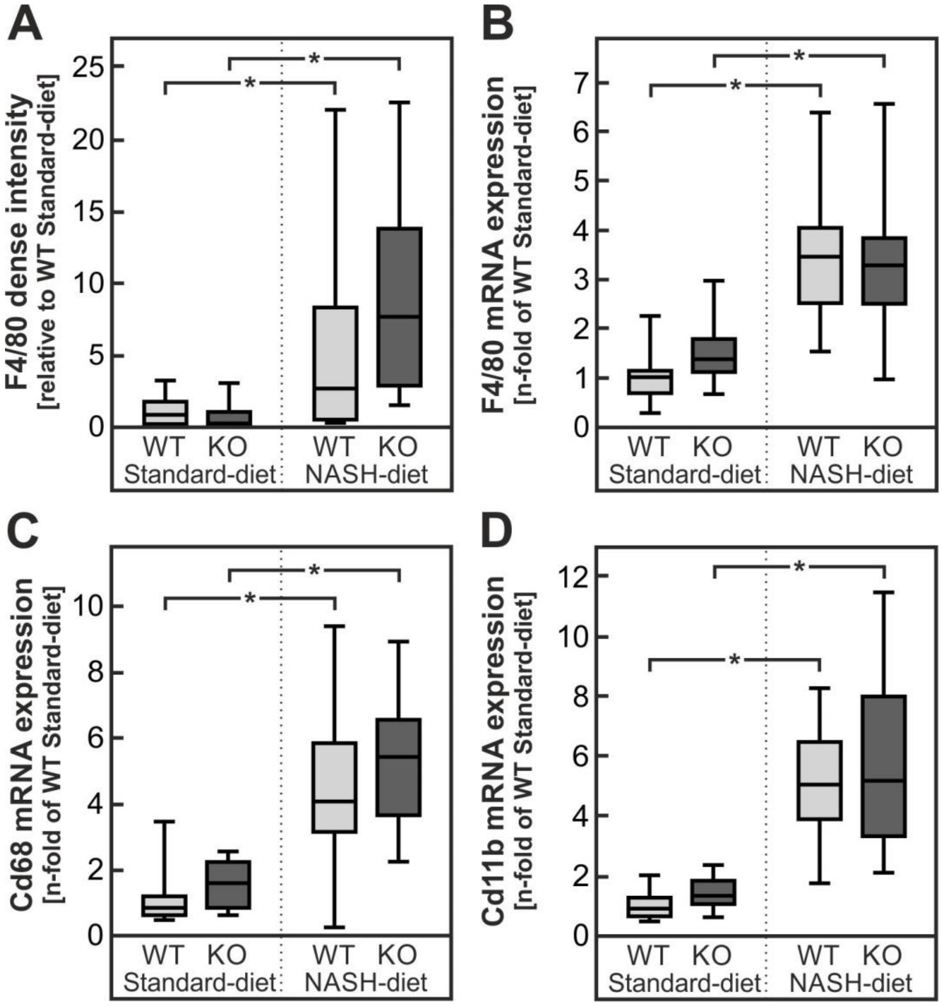

Supplementary Figure S1: Determination of macrophage infiltration in livers of wild-type and mPGES-1-deficient mice fed a standard- or NASH-diet. Male mPGES-1$^{+/+}$ (WT) or mPGES-1$^{-/-}$ (KO) mice received the diets for 20 weeks. (**A**) Quantification of F4/80-stained microphotographs calculated by dense intensity of F4/80 relative to the number of hepatocyte nuclei per field in 5 randomly chosen microphotographs per liver section. (**B**, **C**, **D**) Relative mRNA expression of F4/80 (gene name *Adger*), Cd68 and Cd11b (gene name *Itgam*). Values are median (line), upper- and lower quartile (box) and extremes (whiskers) in 20-25 samples (**A**) or in 12-28 mice per group (**B**, **C**, **D**). Statistics: Two-way-ANOVA with Tukey's post hoc test for multiple comparisons. *: $p < 0.05$.

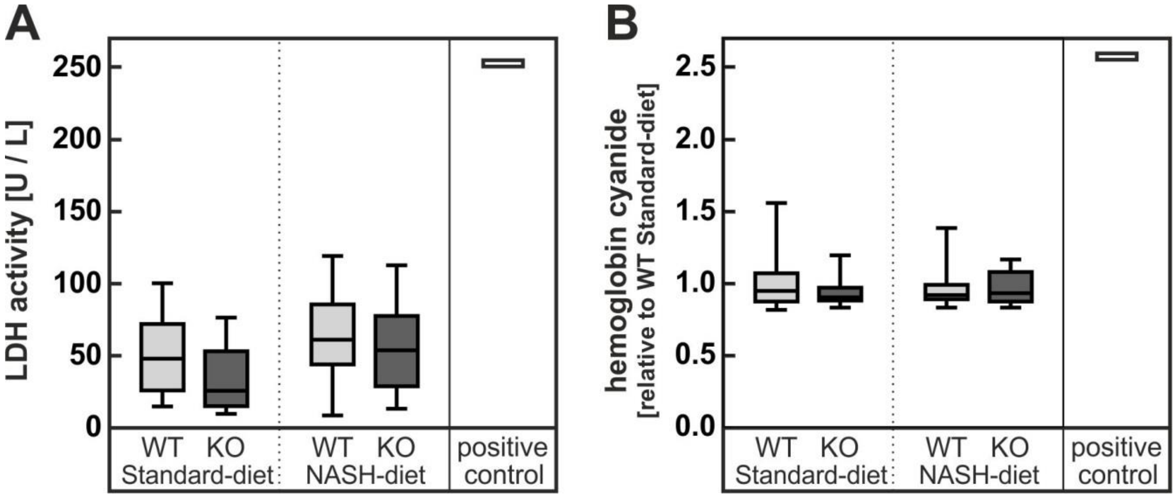

Supplementary Figure S2: LDH activity (A) and hemoglobin cyanide (B) levels in serum of wild-type and mPGES-1-deficient mice fed a standard- or NASH-diet. Male mPGES-1$^{+/+}$ (WT) or mPGES-1$^{-/-}$ (KO) mice received the diets for 20 weeks. Immediately after sacrifice cardiac blood was taken for the analysis of serum parameters. Lactate dehydrogenase (LDH) activity (A) and hemoglobin cyanid (B) were measured in all serum samples used in the animal study for further analysis to exclude hemolysis. Methods were described in the Supplementary Methods section. A hemolytic serum sample was used as a positive control. Values are mean ± SEM of 18-28 mice per group. Statistics: Two-way-ANOVA with Tukey's post hoc test for multiple comparisons, no significant differences between the groups with $p < 0.05$.

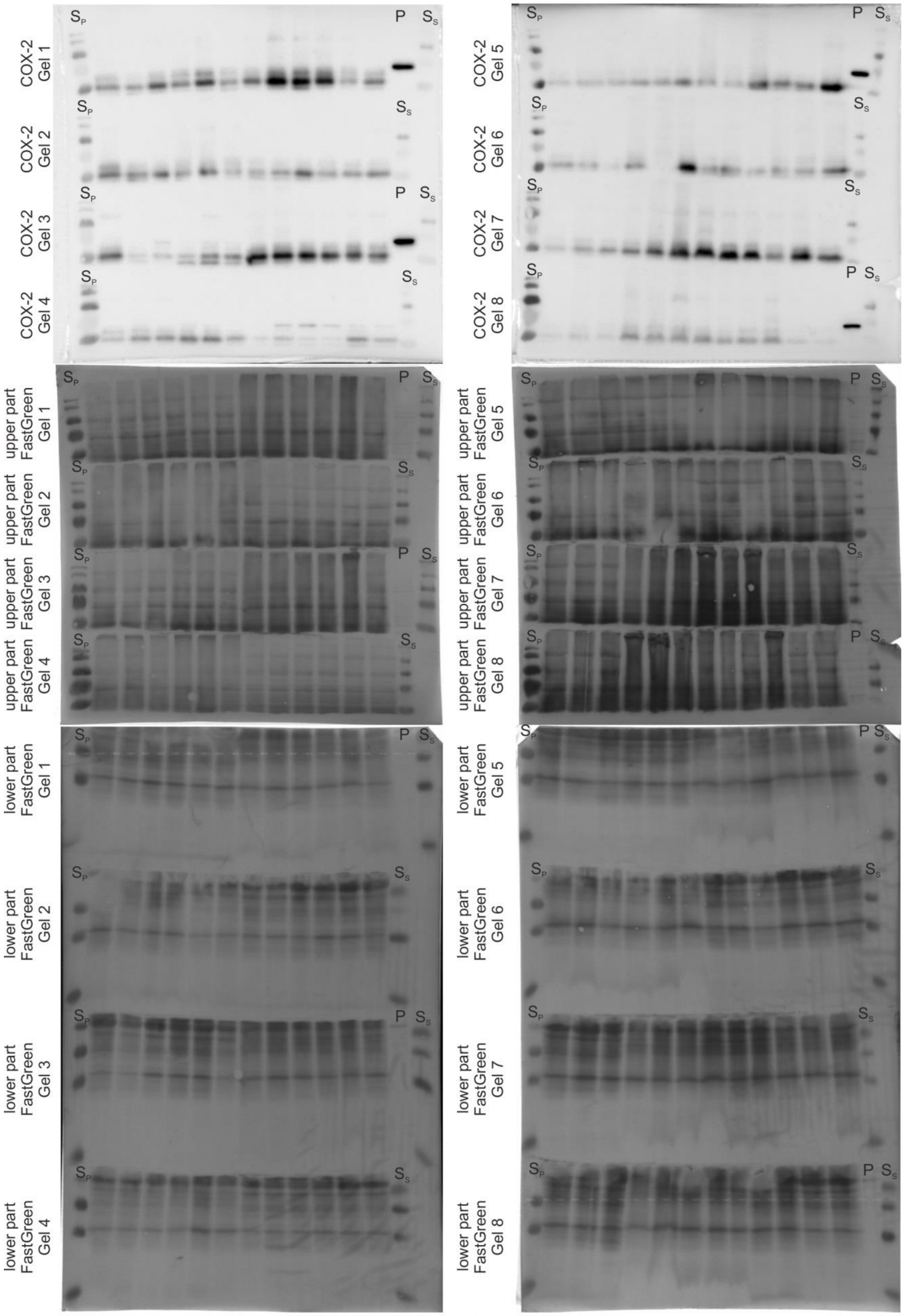

SAMPLE ORDER

Gel 1, Gel 3	Standard-diet		NASH-diet	
	3 x WT	3 x KO	3 x WT	3 x KO
Gel 2, Gel 4	NASH-diet		Standard-diet	
	3 x KO	3 x WT	3 x KO	3 x WT

SAMPLE ORDER

Gel 5	Standard-diet		NASH-diet	
	3 x WT	3 x KO	3 x WT	3 x KO
Gel 6	NASH-diet		Standard-diet	
	3 x KO	3 x WT	3 x KO	3 x WT
Gel 7	Standard-diet	NASH-diet		
	3 x WT	3 x WT	3 x KO	3 x WT
Gel 8	Standard-diet	NASH-diet		Standard-diet
	3 x WT	3 x WT	3 x KO	3 x WT

Supplementary Figure S3: COX-2 protein expression in livers of wild-type and mPGES-1-deficient mice fed a standard- or NASH-diet. SDS-PAGE with 12.5 % acrylamide was performed with 12 liver homogenates per gel in the described sample order. Gels were cut on a horizontal line (about 50 kDa) and gels 1 - 4 or gels 5 - 8 (upper parts with range < 50 kDa, lower parts with range > 50 kDa) were blotted on one membrane. Dense intensity of COX-2 was normalized to FastGreen staining (sum of defined range (180 to 50 kDa and 40 to 20 kDa) from upper and lower part), which was verified on the same Western blot membrane as a loading control. P: homogenate of HEK cells over-expressing human COX-2 as a positive control. COX-2 proteins were detected at about 70 kDa in human cell homogenates and about 60 kDa in mice liver homogenates.

S_P: molecular weight standard "PageRuler Prestained Protein Ladder" (Life Technologies, Darmstadt, Germany), bands with 180, 130, 100, 70, 55, 43, 34, 26 and 17 kDa. S_S: molecular weight standard "Spectra Multicolor Broad Range Protein Ladder" (Life Technologies, Darmstadt, Germany), bands with 260, 140, 100, 70, 50, 40, 35, 25 and 15 kDa.

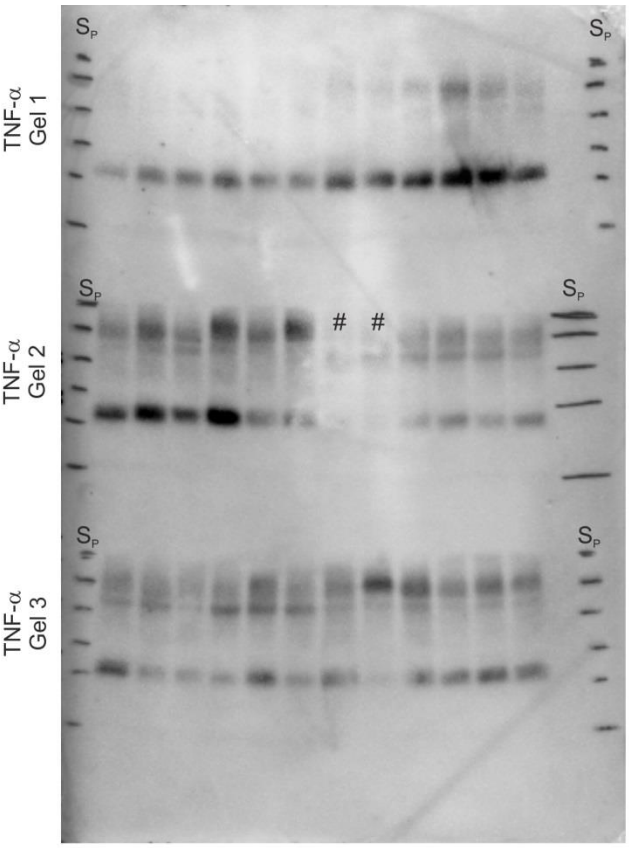

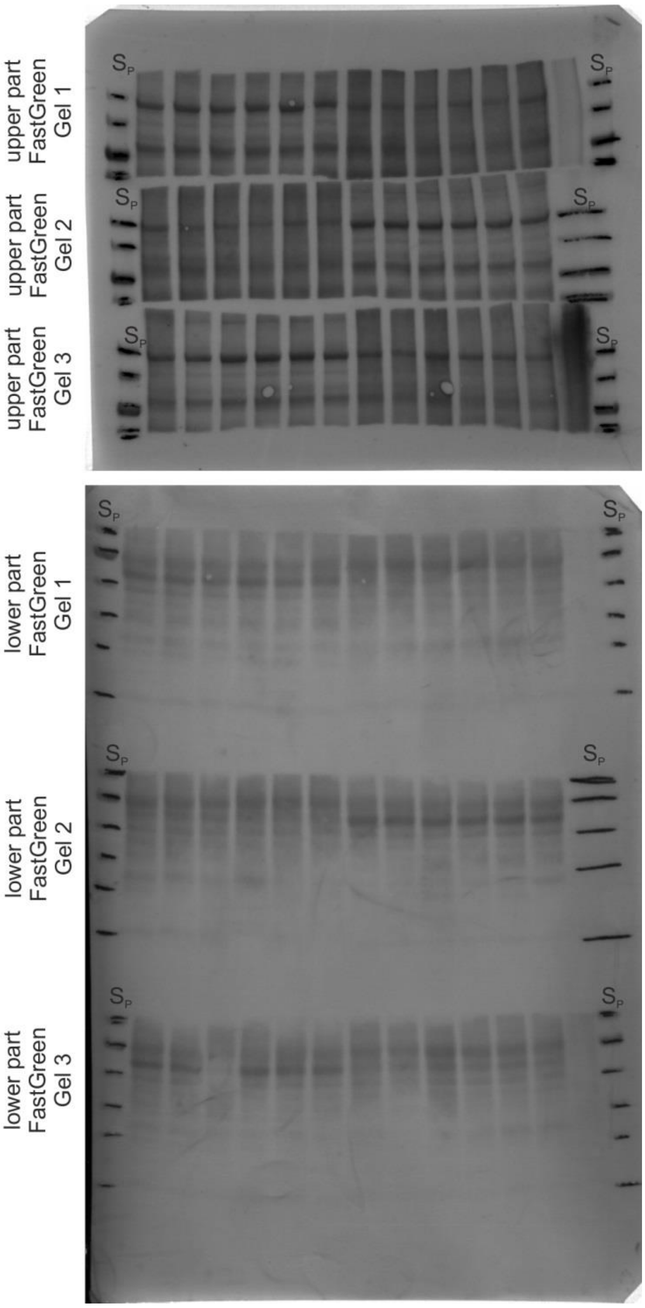

SAMPLE ORDER

	Standard-diet		NASH-diet	
Gel 1, Gel 3	3 x WT	3 x KO	3 x WT	3 x KO
	NASH-diet		Standard-diet	
Gel 2	3 x KO	3 x WT	3 x KO	3 x WT

Supplementary Figure S4: TNF-α protein expression in livers of wild-type and mPGES-1-deficient mice fed a standard- or NASH-diet. SDS-PAGE with 12.5 % acrylamide was performed with 12 liver homogenates per gel in the described sample order. Gels were cut on a horizontal line (about 70 kDa) and 3 gels (upper parts with < 70 kDa, lower parts with range > 70 kDa) were blotted on one membrane. Dense intensity of TNF-α at about 26 kDa was normalized to FastGreen staining (sum of defined range (180 to 70 kDa and 40 to 20 kDa) from upper and lower part), which was verified on the same Western blot membrane as a loading control. S_P: molecular weight standard “PageRuler Prestained Protein Ladder” (Life Technologies, Darmstadt, Germany), bands with 180, 130, 100, 70, 55, 43, 34, 26 and 17 kDa. #: lanes were excluded from calculation.

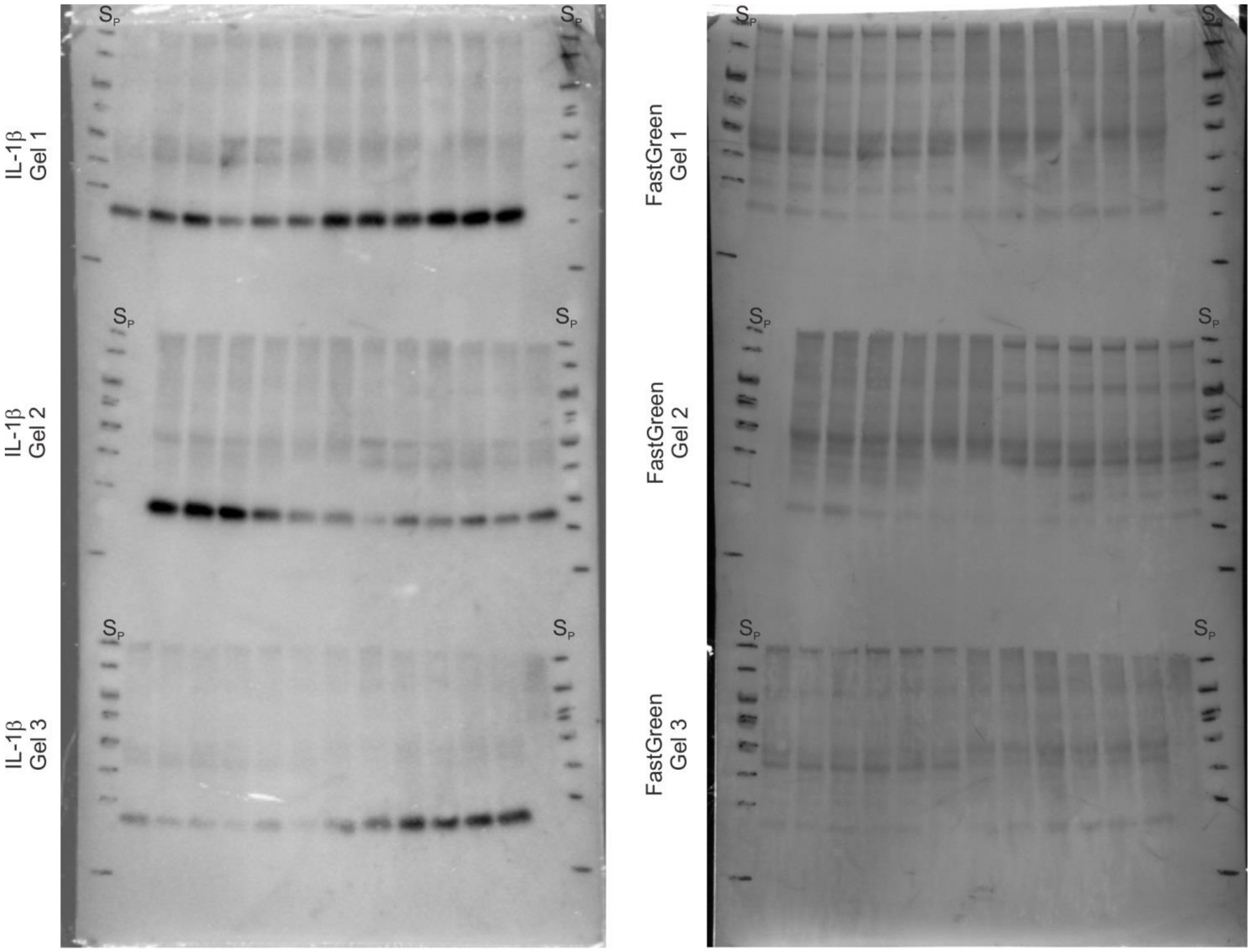

SAMPLE ORDER

	Standard-diet		NASH-diet	
Gel 1, Gel 3	3 x WT	3 x KO	3 x WT	3 x KO
	NASH-diet		Standard-diet	
Gel 2	3 x KO	3 x WT	3 x KO	3 x WT

Supplementary Figure S5: IL-1β protein expression in livers of wild-type and mPGES-1-deficient mice fed a standard- or NASH-diet. SDS-PAGE with 12.5 % acrylamide was performed with 12 liver homogenates per gel in the described sample order. 3 Gels were blotted on one membrane. Dense intensity of IL-1β at about 30 kDa was normalized to FastGreen staining (defined range from about 180 to 40 kDa), which was verified on the same Western blot membrane as a loading control..

S_P: molecular weight standard “PageRuler Prestained Protein Ladder” (Life Technologies, Darmstadt, Germany), bands with 180, 130, 100, 70, 55, 43, 34, 26 and 17 kDa.

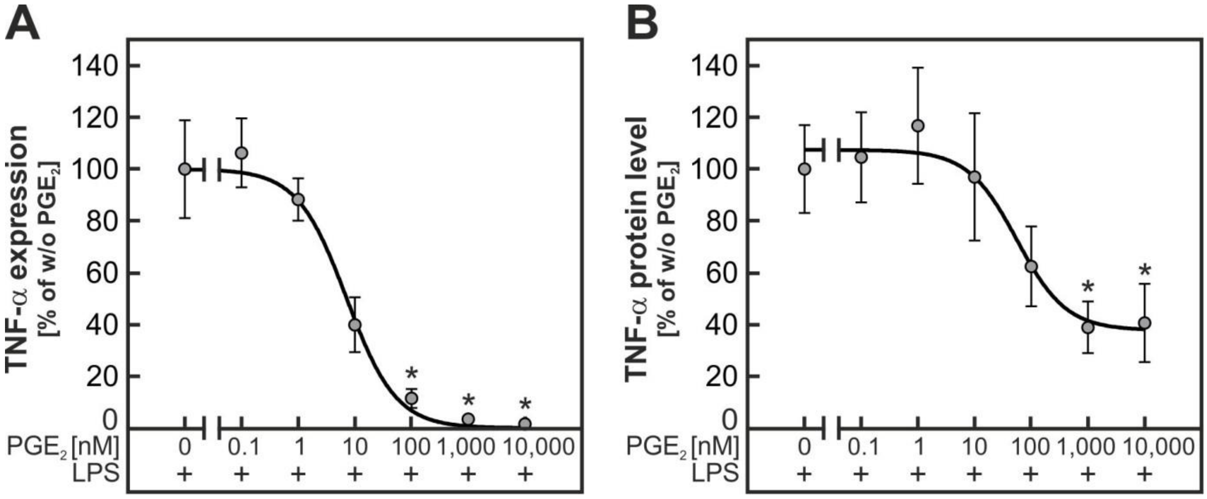

Supplementary Figure S6: Dose response curve of PGE_2-mediated inhibition of LPS-induced TNF-α expression in peritoneal macrophages. Peritoneal macrophages from male mPGES-$1^{+/+}$ (WT) mice were stimulated with 1 ng/mL LPS and indicated concentrations of PGE_2 (Enzo Life Science, Lörrach, Germany) for 24 h. (**A**) Relative mRNA expression of TNF-α was determined by RT-qPCR using Hprt as reference gene. (**B**) Levels of TNF-α were determined in cell culture supernatants. Values are mean ± SEM of 3-4 independent experiments. Statistics: Student's t-test for unpaired samples. *: versus w/o PGE_2 with $p < 0.05$. Calculated IC50 was 7.2 nM PGE_2.

Abbreviations: LPS: lipopolysaccharide; PGE_2: prostaglandin E_2; w/o: without.

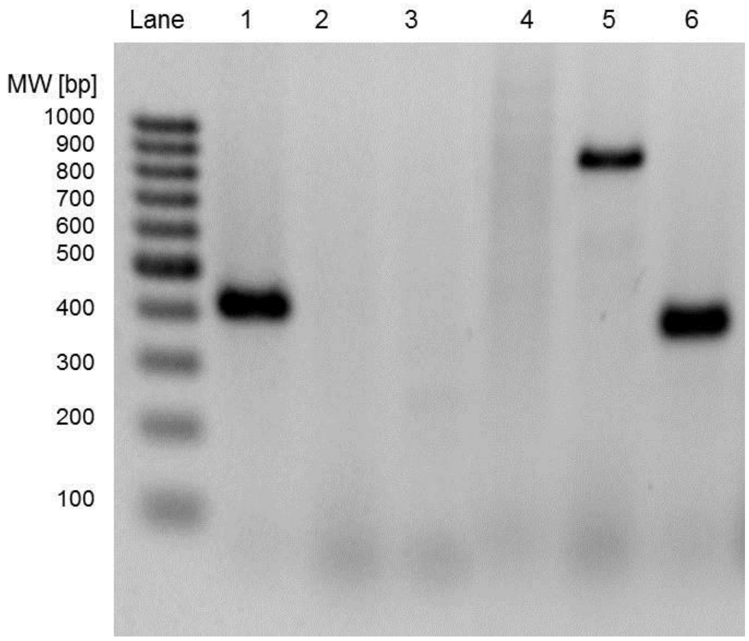

Supplementary Figure S7: Evidence of mPGES-1-deficiency. Genotyping in tail biopsies from mPGES-1$^{+/+}$ (WT, lane 1, 2, 3) or mPGES-1$^{-/-}$ (KO, lane 4, 5, 6) mice. KO mice were deficient in part of exon 1 as well as complete intron 1 and exon 2 of *Ptges* gene. Genomic DNA was isolated and PCR with three sets of oligonucleotides performed: (I) Forward primer in intron 1 and reverse primer in intron 2 resulted in PCR product of 413 bp in WT mice (lane 1) and no PCR product in KO mice (lane 4); (II) forward primer in vector Neo cassette and reverse primer in intron 2 resulted in no PCR product in WT mice (lane 2) and a 720 bp PCR product in KO mice (lane 5); (III) forward primer in vector Neo cassette and reverse primer in vector Neo cassette resulted in no PCR product in WT mice (lane 3) and a 339 bp PCR product in KO mice.

Supplementary Methods

Serum analysis.

LDH activity: Serum was incubated with 100 mM phosphate buffer (pH 7.0) with 0.2 mM NADH. Immediately after addition of sodium pyruvate (end concentration 0.6 mM) absorbance at 339 nm was measured every 30 s ($E_{0\ min}$ – $E_{5\ min}$). LDH activity was calculated with Δ E / min x 5250 U / L as described (MERCK, Klinisches Labor, 1974).

Hemoglobin cyanid levels: Serum was incubated with 2.5 mM phosphate buffer (pH 7.2) with 0.6 mM potassium hexacyanoferrat(III), 1 mM sodium cyanid, 1.5 mM sodium chloride and 0.05% Triton-X as described (MERCK, Klinisches Labor, 1974). After 5 min absorbance at 540 nm was measured. Values were calculated relative to the mean of all values in the WT Standard-diet group.

Quantification of immunohistochemistry analysis.

TUNEL-assay: A macro was designed for automatic analysis of the number of hepatocyte nuclei per field using ImageJ software. The following parameters were defined: 'medianRadius = 2' as well as 'size (pixel^2) = 1000' and 'circularity = 0.25-1.00' for the command 'analyze particles'. Threshold was adjusted to 'threshold = 1-185' for counting all hepatocyte nuclei and 'threshold = 1-160' for counting TUNEL-positive stained hepatocyte nuclei. Results are expressed by ratio of TUNEL-positive stained hepatocyte nuclei relative to all hepatocyte nuclei in % (Figure 4E).

Cleaved caspase 3 immunohistochemistry analysis: Since automatic analysis by ImageJ software in this setup was inaccurate, cleaved caspase 3 positive cells per field were counted manually in masked randomized microphotographs. Values were compared to hepatocyte nuclei per field that were also counted manually in a blinded form (Figure 4F).

F4/80 immunohistochemistry analysis: ImageJ software was used and a macro was designed for automatic analysis of the dense intensity of immunohistochemistry staining of F4/80 using the 'histogram' tool. F4/80-positive areas were defined as sum of histogram data in the range of 120-210. Values were calculated relative to the number of hepatocyte nuclei per field that were counted manually in a blinded form (Supplementary Figure S1).
